# Robust evidence supports a causal link between higher birthweight and longer telomere length: a mendelian randomization study

**DOI:** 10.3389/fgene.2024.1264028

**Published:** 2024-06-21

**Authors:** Zhuoya Zhang, Jiale Zhang, Kaiqi Zhang, Xiaolei Ge, Xu Zhai

**Affiliations:** ^1^ Affiliated Hospital of Nanjing University of Chinese Medicine, Nanjing, China; ^2^ Institute of Basic Theory for Chinese Medicine, China Academy of Chinese Medical Sciences, Beijing, China; ^3^ Wangjing Hospital of China Academy of Chinese Medical Sciences, Beijing, China

**Keywords:** mendelian randomization, birthweight, telomere length, causal relationship, genetic variants

## Abstract

**Background:**

Observational studies have suggested a potential relationship between birthweight and telomere length. However, the causal link between these two parameters remains undefined. In this study, we use Mendelian Randomization (MR). This method employs genetic variants as instrumental variables, to explore the existence of causal associations and elucidate the causal relationship between birth weight and telomere length.

**Methods:**

We used 35 single nucleotide polymorphisms (SNPs) as instrumental variables for birth weight. These SNPs were identified from a meta-analysis involving 153,781 individuals. Furthermore, we obtained summary statistics for telomere length from a study conducted on 472,174 United Kingdom Biobank participants. To evaluate the causal estimates, we applied the random effect inverse variance weighted method (IVW) and several other MR methods, such as MR-Egger, weighted median, and MR-PRESSO, to verify the reliability of our findings.

**Results:**

Our analysis supports a significant causal relationship between genetically predicted birth weight and telomer3e length. The inverse variance weighted analysis results for birth weight (Beta = 0.048; 95%CI = 0.023 to 0.073; *p* < 0.001) corroborate this association.

**Conclusion:**

Our study provides robust evidence supporting a causal link between higher birth weight and longer telomere length.

## Introduction

Birthweight is an important predictor of fetal health and has been associated with various health outcomes later in life, including type 2 diabetes, cardiovascular disease, and cancer (Cheng et al., 2021; Hu et al., 2022). Telomeres are the protective caps at the ends of chromosomes implicated in these same health outcomes, with shorter telomeres associated with an increased risk of age-related diseases. Recent studies (Guzzardi et al., 2016; Guyatt et al., 2017) have suggested that birthweight may be related to telomere length, with infants with higher birthweights having longer telomeres. However, whether this relationship is causal or other factors such as maternal age, smoking, or nutrition confound, this association is unclear.

Understanding the causal relationship between birthweight and telomere length has important implications for understanding the biological mechanisms underlying the relationship between birthweight and health outcomes later in life. It may also inform strategies for preventing age-related diseases and improving birth outcomes. Our study will contribute to the growing literature investigating the relationship between birthweight and telomere length. It may provide insight into potential interventions to improve birth outcomes and prevent age-related diseases.

**FIGURE 1:**
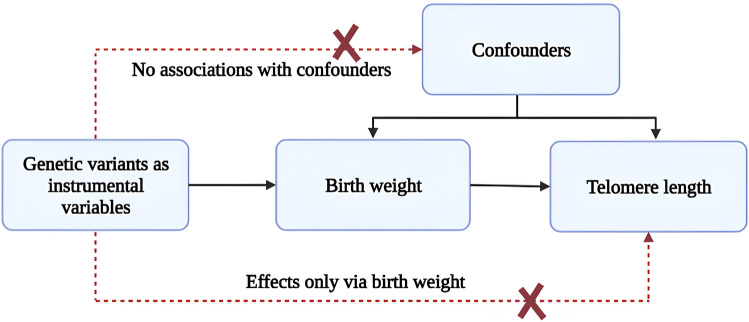
Mendelian randomization analyses to investigate associations between birth weight and telomere length. The broken lines signify potential causal effects (pleiotropic or direct) between variables that would violate the assumptions of Mendelian randomization.

This study uses Mendelian randomization, a powerful tool for causal inference, to Figure 1 illustrates the Mendelian randomization analyses conducted to explore associations between birthweight and telomere length. Mendelian randomization uses genetic variants as instrumental variables to test for causal effects of an exposure on an outcome, allowing for more robust conclusions about causality than traditional observational studies. Using genetic birthweight predictors as instrumental variables, we aim to assess whether higher birthweight is causally related to longer telomere length. This study aims to use Mendelian randomization to investigate the causal relationship between birthweight and telomere length, and to provide insight into the biological mechanisms underlying the relationship between birthweight and health outcomes later in life. By providing evidence for a causal relationship between birthweight and telomere length, our study may inform strategies for improving birth outcomes and preventing age-related diseases.

## Materials and methods

### Study design

This investigation explored the potential association between birth weight and telomere length utilizing a two-sample Mendelian randomization (MR) approach. We adhered to the STROBE-MR Statement guidelines (Skrivankova et al., 2021), and utilized summary data from previously ethically-approved public genome-wide association studies (GWAS).

### Instrument selection for birth weight

GWAS summary statistics for birth weight were drawn from a comprehensive meta-analysis of 153,781 individuals, unearthing 60 loci with significant fetal genotype-birth weight associations (*p* < 5 × 10^−8^). These single nucleotide polymorphisms (SNPs) formed the genetic instruments for the ensuing MR analysis. We subsequently curated 35 SNPs with robust birth weight associations at a genome-wide significance level (*p* < 5 × 10^−8^), ensuring independence through a linkage disequilibrium (LD) test (r2 < 0.001 and distance >10,000 kb) (Horikoshi et al., 2016). Palindromic SNPs with intermediate allele frequencies were excluded to prevent possible inversion of causal effect direction. For missing SNPs in the outcome data, we utilized substitute proxy SNPs, identified through SNIPA. A manual screening using PhenoScanner was performed to preclude associations with confounders. Manual screening using PhenoScanner ensured no association with confounders (Kamat et al., 2019). For initial analysis, these 35 independent SNPs served as instrumental variables. The proportion of birth weight variance accounted for by each SNP was evaluated using R2, with the instrumental variables’ effectiveness gauged via F statistic, where an F value exceeding ten was indicative of strong instruments.

### Genetic associations with telomere length

The GWAS summary statistics for telomere length were obtained from a study involving 472,174 United Kingdom Biobank participants, identifying 197 independent sentinel variants across 138 genomic loci associated with telomere length (Codd et al., 2021).

### Statistical analysis

We conducted MR analyses utilizing R software version 4.2.2 and relied on the MendelianRandomization, EasyMR, and MR-PRESSO packages for these analyses. The primary MR analyses were conducted using inverse variance weighted (IVW) analyses, with the heterogeneity of IVW estimates assessed through the Cochran Q test (significance level: *p* < 0.05).

To ensure the robustness of our results, we also performed several sensitivity analyses employing different MR methods: MR-Egger, weighted median, simple mode, weighted mode, and MR-PRESSO. The MR-Egger method, a prominent approach for detecting and correcting pleiotropy, was utilized to estimate the potential impact of pleiotropy on the results. An MR-Egger intercept with a *p*-value of less than 0.05 indicates the presence of directional pleiotropy, which may result in biased MR estimates (Burgess and Thompson, 2017). The weighted median approach was utilized to generate a more reliable estimate even when up to 50% of the instruments were invalid, by taking the median of all possible pairwise IVW estimates. Furthermore, two additional MR methods, Simple and Weighted modes, were employed to further strengthen the findings’ validity. The Simple mode selects SNPs based on their individual association with the exposure and outcome. In contrast, the Weighted mode selects SNPs based on their overall strength of association with the exposure and outcome (Bowden and Holmes, 2019). These methods provide alternative instrumental variable selection criteria and estimation techniques. Additionally, the MR-PRESSO method, which detects and corrects for outliers and horizontal pleiotropy, was employed to assess the potential impact of outliers on the results (Verbanck et al., 2018), we also use RadialMR to identify outliers. RadialMR is a Mendelian randomization method that improves the accuracy of causal effect estimates by projecting weak instrumental variables onto a vector that maximizes the variance of the exposure variable (Bowden et al., 2018). It can be applied to high-dimensional genetic data and is useful for large-scale MR studies. Each method allowed us to estimate the beta coefficients, which indicate the predicted change in telomere length for each one-unit increase in birth weight, assuming that all other variables remain constant.

## Results

**FIGURE 2:**
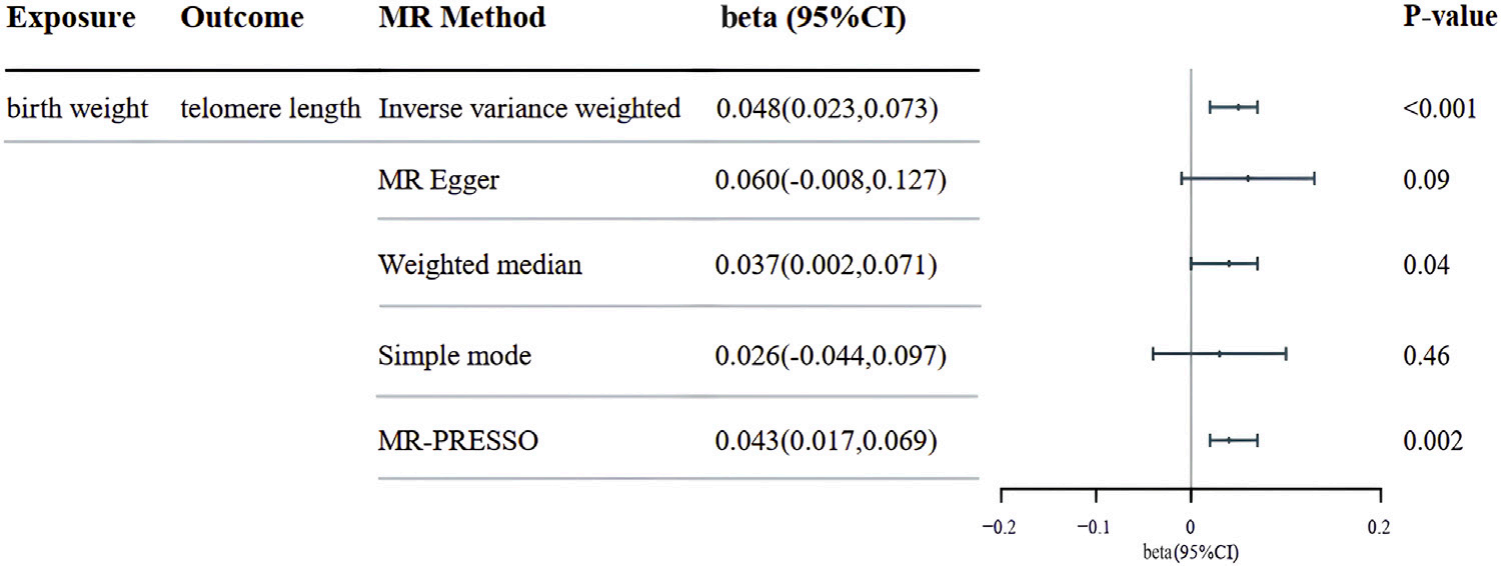
The beta depicting the associations between genetically predicted birth weight and telomere length are presented. CI, confidence interval; MR, Mendelian randomization.

Our MR analysis utilizing the inverse variance weighted (IVW) method revealed a significant association between genetically predicted birth weight and leukocyte telomere length (beta = 0.048; 95%CI = 0.023 to 0.073; *p* < 0.001). This effect was consistently observed across most Mendelian randomization methods, as illustrated in Figure 2. These findings underscore the importance of birth weight as a potential determinant of telomere length, which has far-reaching implications for understanding the genetic regulation of cellular aging and associated diseases. Our results were unlikely confounded by heterogeneity or pleiotropic effects, as demonstrated by the absence of significant heterogeneity of IVW estimates (Q-test, *p* > 0.83) and directional pleiotropy (MR-Egger intercept, *p* > 0.72).

## Discussion

### Mechanism

The causal relationship detected through our Mendelian randomization approach indicates that higher birthweight *per se* may promote telomere lengthening through biological pathways. This challenges the traditional view that the association is largely confounded. Usually, we think that the weight gain may be related to the short telomeres. Researchers from the University of Hasselt in Belgium found that (Martens et al., 2016) a significant inverse relationship between maternal pre-pregnancy BMI and newborn telomere length, for every one-unit increase in BMI, infant telomeres shortened by an estimated 50 base pairs after adjusting for various maternal and newborn characteristics. A cohort study (Wei et al., 2021) from China also found a relationship between prematernal overweight and shorter neonatal telomeres. The specific mechanisms still require elucidation but could involve pathways influencing metabolism, growth factors, and inflammation (Movérare-Skrtic et al., 2009; Xu et al., 2014; Lv et al., 2022).

Several mechanistic hypotheses can be proposed based on current knowledge. Higher birth weight likely reflects greater fetal nutrition, which can influence multiple biological processes relevant to telomere maintenance.

For example, higher birthweight may be associated with increased production of growth factors like insulin-like growth factor 1 (IGF-1) (Alekseenkova et al., 2022). IGF-1 has been shown to activate telomerase and protect telomeres from shortening (Barbieri et al., 2009). Higher IGF-1 levels during critical periods of prenatal development could therefore program longer telomeres that persist into adulthood (Kaplan et al., 2009). High IGF-1 may be an independent predictor of longer leukocyte telomere length.

In addition, greater fetal nutrition may reduce oxidative stress and inflammation, which are known to damage telomeres and accelerate their shortening (Wang et al., 2021). A higher mean TNF-α/IL-10 ratio during pregnancy was significantly associated with a shorter neonatal telomere length (Lazarides et al., 2019). Higher antioxidant defenses and lower levels of pro-inflammatory cytokines in heavier newborns could provide a protective environment for telomeres (Flannagan et al., 2020). However, more research is needed to clarify the role of oxidative stress and inflammation.

Higher birthweight may also be linked with metabolic changes that influence telomere biology. Heavier babies tend to have higher insulin sensitivity, adiponectin levels and a more favorable metabolic profile that extends into later life (Fall 2013; Van Hulst et al., 2018). These metabolic factors could potentially reduce age-related telomere attrition through poorly understood pathways.

On a cellular level, higher birthweight may promote proliferation of cells with longer telomeres during critical periods of development (de Zegher et al., 2017). Telomere length is known to vary between different cell types. Heavier babies may experience preferential expansion of cell populations with initially longer telomeres, programming their overall telomere profile. But this hypothesis requires further investigation.

Finally, epigenetic modifications like DNA methylation and histone modifications could mediate the effects of birthweight on telomere dynamics (Madden et al., 2021). Early life exposures determining newborn size may induce epigenetic changes that persistently alter telomere maintenance gene expression (Sibert et al., 2021). Recent studies (Marioni et al., 2018; Cerveira de Baumont et al., 2021; Shinko et al., 2022; Robinson et al., 2023) have found that epigenetic age serves as a precise metric for quantifying the subtle yet significant alterations in DNA methylation patterns that naturally accompany the passage of time or are associated with various biomarkers indicative of the aging process. These changes, which often manifest as alterations in the methylation status of specific genes or genomic regions, are not encoded in the DNA sequence itself but rather reflect external influences and internal biological processes. For instance, telomere length, a well-known biomarker of cellular aging, is closely linked to epigenetic age, indicating a synergistic relationship between the two phenomena. Importantly, epigenetic age has emerged as a robust predictor of both morbidity and mortality, surpassing even chronological age in its ability to forecast health outcomes. By providing insights into the dynamic interplay between genetic and environmental factors that shape biological aging, epigenetic age offers a valuable tool for understanding the complex mechanisms underlying age-related diseases and conditions. However, epigenetic mechanisms have not been extensively studied in this context. Future studies leveraging -omics technologies, cellular models, and pathway analyses may provide novel mechanistic insights and identify potential targets for intervention (Zhang et al., 2017).

### Clinical relevance

If confirmed in future studies, the causal relation suggests interventions to improve birthweight and optimize fetal growth may help lengthen telomeres and delay age-related diseases. However, a large effect on clinical outcomes is uncertain and targeted telomere-lengthening strategies are not yet recommended.

Our findings imply that promoting healthy fetal growth and optimizing birthweight through potential interventions in pregnancy may help newborns attain longer telomeres that partially protect against age-related disease risks (Entringer et al., 2011). Heavier babies with relatively longer telomeres at birth may enter childhood and adolescence with a cellular advantage that persists into adulthood. However, the true clinical significance remains unclear. Currently, limited evidence directly connects telomere length changes driven by birthweight with meaningful reductions in disease burden and mortality (Needham et al., 2015). More definitive data are needed before birthweight optimization can be firmly recommended as a clinical or public health strategy to lengthen telomeres and delay aging (Fasching, 2018).

While longer telomeres are generally viewed as favorable and associated with better health outcomes, the role of birthweight-related differences in telomere length remains poorly understood. Other factors influencing early life development and health may have a greater impact on lifespan and susceptibility to age-related disease than telomere biology alone (Chen et al., 2022).

Even if purposefully lengthening telomeres through interventions targeting birthweight and fetal growth does translate to some clinical benefits, the magnitude of the effect is likely small compared to other determinants of health over the lifespan (Blackburn et al., 2015). Longitudinal studies quantifying the independent contribution of birthweight-driven telomere lengthening to disease risk and mortality are critically needed.

In the absence of such data, targeted strategies aimed at purposefully lengthening telomeres cannot be recommended based primarily on birthweight. General measures to ensure adequate fetal nutrition and reduce complications in pregnancy may represent the extent of justifiable clinical implications at this time (Daneels et al., 2021; Godhamgaonkar et al., 2021; Córdova-Oriz et al., 2022).

While our findings suggest a potential avenue for delaying aging, robust evidence directly linking birthweight-related differences in telomere length to meaningful gains in healthspan and lifespan is currently lacking (Aviv et al., 2006). Large, long-term studies quantifying the independent contribution of telomere lengthening to clinical outcomes are needed before birthweight optimization or targeted telomere interventions can be recommended for broader application.

### Generalizability

While the findings implicate potential causality, they likely apply primarily to term births within the range studied. The generalizability to other populations, levels of birthweight, and periods remains unclear and requires investigation in diverse samples.

The generalizability of our results depends on several factors. First, our sample consisted primarily of term births within the normal to high birthweight range. The findings may not be directly applicable to preterm infants born at extremely low birthweights (Belfort et al., 2021). Telomere biology and the relationship with birthweight could differ substantially for these high-risk groups. Dedicated studies are needed in preterm and very low birthweight cohorts.

Second, our population was relatively homogenous in terms of ethnicity and socioeconomic status. Generalizability to diverse racial and ethnic groups with varying environmental exposures remains uncertain (Thierry, 2020). Biological and socioeconomic factors could moderate the association between birthweight and telomere length in important ways across different populations (Gebreab et al., 2016; Thayer et al., 2023). Comparative studies in non-Western samples and underserved communities are particularly warranted.

Third, we assessed telomere length at a single timepoint in adulthood. The ability to generalize our findings to other periods of life, particularly early childhood when telomeres are more dynamic, is limited. Studies following individuals from birth and serially measuring telomere lengths are required to establish the full lifespan trajectory (Li et al., 2017). Fourth, genetic variation could influence generalizability across racial/ethnic groups. Different alleles distributions influencing birthweight and telomere biology may modify the observed association in distinct populations (Tyrrell et al., 2012). Future studies incorporating genetic data may help determine populations that are more or less susceptible to the effects of birthweight on telomere length.

Fifth, socioeconomic factors largely unmeasured in our study likely impact both birthweight and telomere maintenance over the lifespan. The generalizability of our findings to populations facing diverse social and economic adversities warrants separate investigation.

Finally, time trends in medical care, nutrition, and environmental exposures may influence the relevance of birthweight for telomere dynamics in future generations (Lai et al., 2023). Continued research in contemporary and longitudinal cohorts will be needed to establish the timeliness of our conclusions.

In summary, while our results provide novel causal evidence in the population studied, considerably more research is warranted to determine the true generalizability across diverse populations, time periods, and levels of birthweight abnormality. Future studies incorporating genetic and socioeconomic data, as well as following individuals from birth into late life, represent critical next steps for establishing the implications across diverse communities (Martens et al., 2020).

### Meaning

Our Mendelian randomization study provides novel causal evidence that higher genetically predicted birthweight leads to longer telomere length.

However, our study still has certain limitations. First, the genetic variants associated with birthweight only explain a small fraction of its heritability. Longer telomeres are not a simple protective factor, as various intrinsic and extrinsic factors influence them. Residual confounding or pleiotropy could partly account for the observed relationship with telomere length. Second, we assumed a linear association between genetically predicted birthweight and telomere length. In reality, the relationship could be more complex at different birthweight percentiles. Our results may primarily reflect associations within the normal birthweight range. Third, telomere length is influenced by many genetic and environmental factors across the lifespan. Birthweight represents just one period that could influence telomere dynamics. Other factors not captured in our analysis may also contribute. Fourth, a significant drawback of MR is the potential influence of horizontal pleiotropy, which can lead to biased causal estimates. However, the consistency of our results with those obtained from alternative MR methods, which are less susceptible to horizontal pleiotropy, lends credence to our findings. Finally, our analyses were restricted exclusively to individuals of European ancestry, thus, our findings might not be extrapolated to other ancestry populations.

## Conclusion

In summary, while our study provides some causal evidence, the limitations indicate that a cautious interpretation is warranted. Replication in larger studies leveraging multiple genetic instruments and complementary approaches will be important to validate and refine our findings. Further research should explore potentially nonlinear and context-dependent effects of birthweight on telomere length over the life-course.

## Data Availability

The original contributions presented in the study are included in the article/supplementary material, further inquiries can be directed to the corresponding author.
